# The Brotherhood Medical Center: Collaborative Foundation of Maternity and Children’s Healthcare Facility for Displaced Syrians

**DOI:** 10.3389/fpubh.2018.00108

**Published:** 2018-04-18

**Authors:** Rahma Aburas, Amina Najeeb, Laila Baageel, Tim K. Mackey

**Affiliations:** ^1^Joint Masters Degree Program in Health Policy and Law, University of California, San Diego, San Diego, CA, United States; ^2^Brotherhood Medical Center for Women and Children, Atimah, Syria; ^3^Department of Anesthesiology, University of California, San Diego School of Medicine, San Diego, CA, United States; ^4^Division of Global Public Health, Department of Medicine, University of California, San Diego School of Medicine, San Diego, CA, United States; ^5^Global Health Policy Institute, San Diego, CA, United States

**Keywords:** maternal child health, Syrian crisis, humanitarian health aid, internally displaced people, conflict and health

## Abstract

The United Nations has declared the Syrian conflict, with more than 50% of Syria’s population currently displaced, as the worst humanitarian crisis of the twenty-first century. The Syrian conflict has led to a collapse of infrastructure, including access to critical and lifesaving healthcare services. Women and children account for approximately 75% of internally displaced Syrians and refugees. This population is also particularly vulnerable to poor health outcomes, a condition worsened by lack of access to maternal and child health services. In response to this crisis, a partnership of Saudi and Syrian physicians established a non-profit healthcare facility named the Brotherhood Medical Center (BMC) to serve women and children within a safe area near the Syrian–Turkish border. The project began in September 2014 and was implemented in three phases of establishment, phased construction and formal launch and operation. Currently, the BMC is working at about 70% of its capacity and is run in partnership with the Syrian Expatriate Medical Association. Although there was strong initial support from donors, the BMC continues to face many financial and operational challenges, including difficulties in transferring money to Syria, shortage of medical supplies, and lack of qualified medical personnel. Despite these challenges, the BMC represents a critical model and an important case study of the challenges of delivering healthcare services to underserved populations during an ongoing conflict. However, more robust support from the international community is needed to ensure it continues its important health and humanitarian mission.

## Introduction

The ongoing Syrian civil war now constitutes the worst humanitarian crisis in modern history, with the Syrian Center for Policy Research estimating the death toll at some 470,000 (1). This equates to 11.5% of the entire Syrian population either being killed or injured as a result of a conflict that began more than 7 years ago and which shows no end in sight (2, 3). This massive upheaval has created 7.6 million internally displaced people (IDP) who continue to reside within Syria coupled with approximately 4.8 million Syrian refugees who have fled to neighboring countries to seek sanctuary, equating to one the world’s largest displacement crises (4).

Importantly, this conflict has severely impacted the most vulnerable population group: women and children. Before the conflict, women in Syria enjoyed access to standard maternal and antenatal healthcare with 96% of women delivering at home or in hospitals attended to by a skilled birth attendant (5). Post-conflict, coverage for critical maternal and child health (MCH) services has rapidly deteriorated, with Syrian women and their children now experiencing low-birth weight, antenatal complications, premature labor, poor pregnancy outcomes, and increased puerperal infections (3). Lack of adequate access and funding to MCH services has also resulted in higher rates of unwanted pregnancies due to absence of family planning support (6).

Remaining healthcare facilities that have not been destroyed due to systematic military targeting largely prioritize victims with severe and acute injuries among IDP and refugee populations (2). This has resulted in the de-prioritization of MCH services that are now afforded less attention within a Syrian health system already crippled by war, leaving women and children in desperate need for other medical and humanitarian access points for services previously provided by the public health system (7). Hence, there is an immediate and acute need for medical humanitarian intervention to fill this gap in MCH services.

## Background: “Birth” of the Brotherhood Medical Center (BMC)

**Figure 1:**
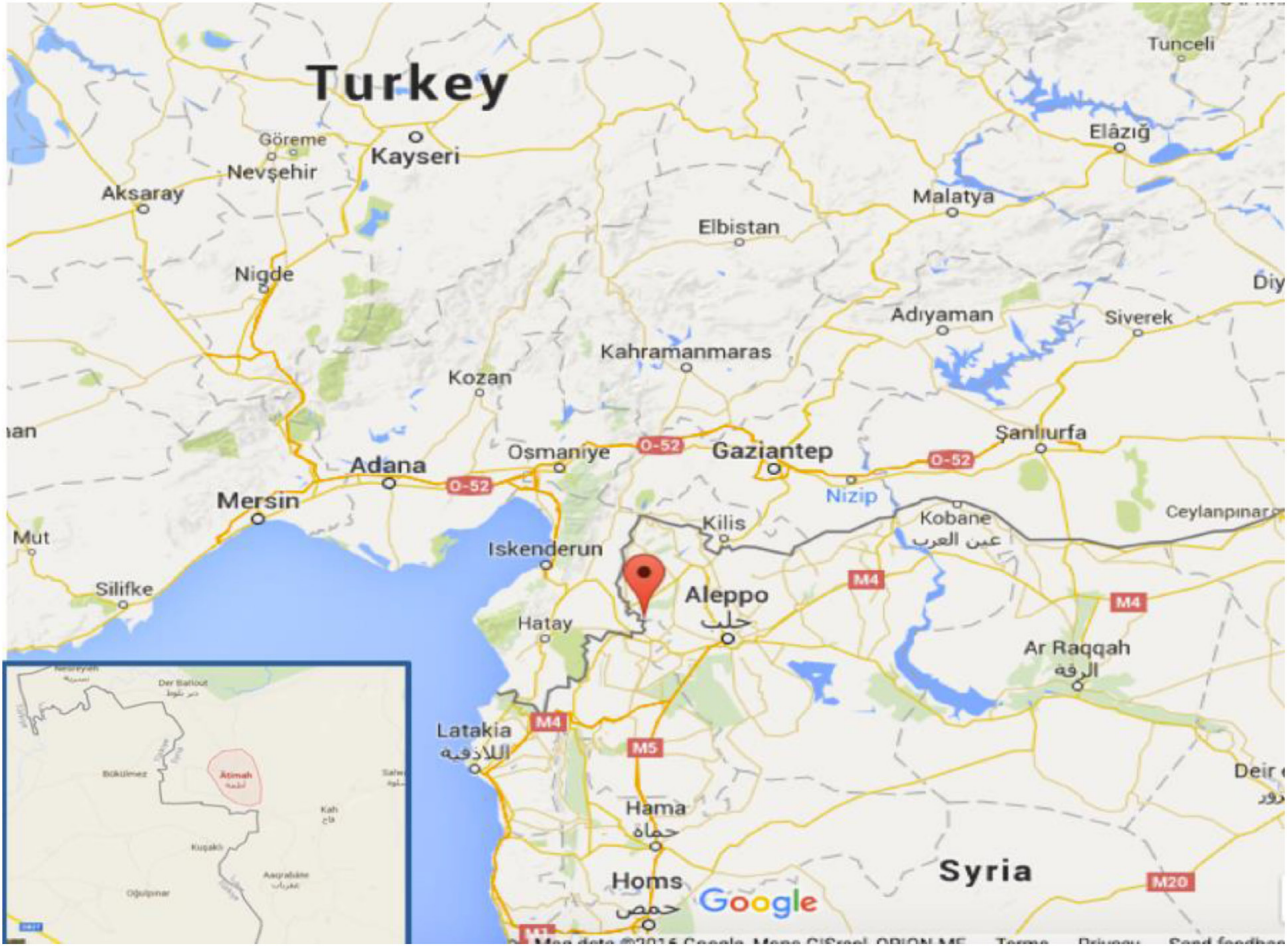
Syrian–Turkish boarder, map depicting the location of Atimah where the Brotherhood Medical Center is operating (Source: Google Maps).

In view of the urgent need for medical humanitarian aid for Syrian IDPs and in an effort to improve access to MCH services, a group of Saudi physicians and other donors, in collaboration with a group of Syrian physicians, established a healthcare facility named the BMC. The BMC facility was built in a small town named Atimah located on the Syrian side of the Syrian–Turkish border, adjacent to the city of Antakya (see Figure 1) and has the primary focus of serving Syrian women and children. Atimah has an area of approximately 65 square kilometers, had a population of 250,000 people prior to the conflict, and is an area with limited development or infrastructure.

Due to violence occurring during the conflict (including bombing campaigns against unarmed civilians), Syrians had no choice but to escape and settle to certain safety zones (8). The town of Atimah and surrounding areas are generally considered safe, with the ongoing internal displacement of Syrians leading to a continuous influx of Syrian families into the area, raising the population of the town to approximately a million people. The majority of these IDPs (who mostly comprise children, women, and the elderly with intensive healthcare needs) live in camps that lack the basic infrastructure needed to house people for long-term periods (8).

The first iteration of the BMC clinic was established in the heart of the Atimah displacement camps and consisted of only a stethoscope, a chair for sitting in the shadow of a tree, a table, and a box containing common essential drugs. This rudimentary clinic eventually grew into a large canvas tent separated by a curtain into male and female sections, each section served with a volunteer physician of the same gender. This “medical tent” was the only source for healthcare services provided for IDPs residing in the camp and also served the dual purpose of providing basic safety, protection, healing, and shelter. With the continued influx of displaced people and expansion of the Atimah IDP camps, the medical tent was then converted into a small healthcare center composed of several caravans and served permanently by two physicians and a nurse.

In relation to MCH services, obstetrical deliveries took place outside of the medical tent at the camp and were instead provided at the house of one of this study’s coauthors who is a Syrian female physician. Our coauthor estimates that she conducted up to five deliveries per day in her house outside of the camp. Another coauthor of this study, a Saudi female physician, heard about the Atimah camp healthcare center from colleagues. She then decided to visit the site and develop and implement a plan to mobilize donors, enlist the help of medical volunteers, and begin the process of establishing a permanent medical center in Atimah to cover the urgent and growing needs of Syrian women and children IDPs in the area.

### Phased Construction, Launch, and Operation of the BMC

Following mobilization of necessary stakeholders and the generation of capital/donations needed to scale up services in Atimah, a construction and launch plan for the BMC was finalized. The project’s initial construction and operation was supervised by two committees, one in Saudi Arabia and one in Syria with different governance and operational functions (see Box 1). This governance structure allowed the project to have dual oversight from the perspective of both the community of donors funding the project and local stakeholders who would both operate and benefit from access to the BMC’s healthcare services. It also allowed for shared leadership and partnership on short- and long-term planning of the clinic’s design, construction, and operational aspects to ensure it would meet the acute needs of the Syrian IDP population. In addition to the formation of a Saudi–Syrian governance committee structure, the actual operation of the BMC project was implemented in three steps including construction, initial operational phase, and a secondary operational phase as described below.

Box 1 **Governance Structure of Brotherhood Medical Center Project.**

Role and functions of the Saudi committee:

1. Supervise the project and supply donors with updated reports on the progress of the project documented with pictures and other information as follows:
  - Documentation of land ownership instruments and placing ownership under a charity’s name.
  - Follow-up on the construction and operation of the facility from breaking ground, construction milestones, and until completion of project readiness.
  - Formalizing agreement with a local contractor for the project cost and duration.
2. Ensuring appropriate disbursement of funds donated based on documentation and invoices.
3. Monitoring the need for tools, equipment, supplies, drugs, and medical devices for clinical operation of the clinic.

Role of the committee in Syria:

1. Monitoring the day-to-day work and construction of the project, issuing progress reports on construction and operation, and providing information on ongoing needs and suggested changes/modifications to the operational plan to the Saudi committee.
2. Contracting with different local employees according to work needs and supervising the operation of the clinic after the full accomplishment of the project.

#### Phase I: Construction

**Figure 2:**
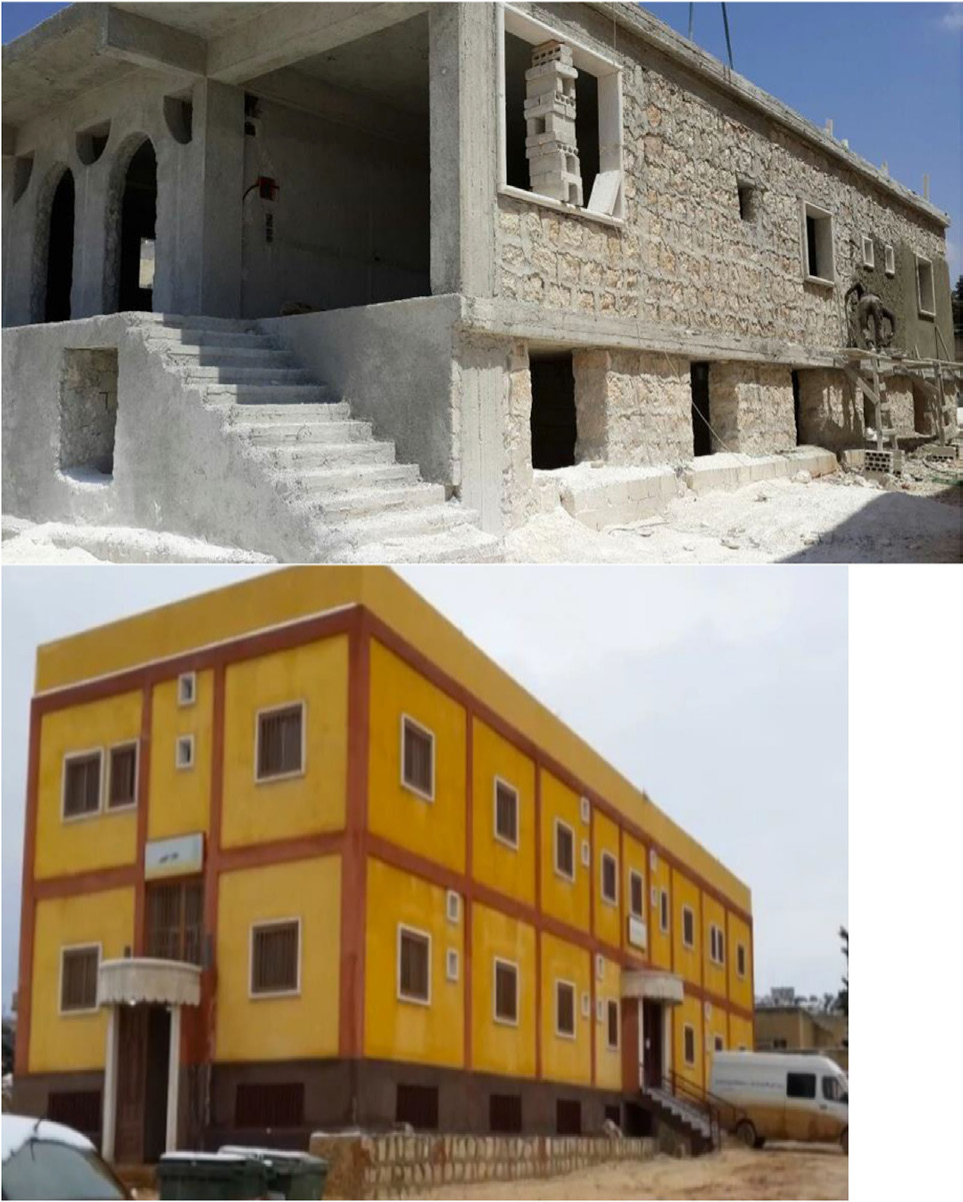
Picture of Brotherhood Medical Center (BMC) at Initial Phase of Construction and BMC Main Hospital Building Post-Construction.

On August 2013, a land parcel of 734 m^2^ was bought from a local landowner, and the building design maps were prepared for initial construction (see Figure 2). The main medical facility composed of a two-story building with a basement floor (Figure 2). The basement contained nine rooms, distributed for the following functions: Physiotherapy (one room), medical warehouse (one room), kitchen (one room), laundry room (one room), and empty rooms (five rooms). The empty rooms were reserved for future use for general surgery and ear surgery. The first floor of the facility primarily serves as the Women & Obstetrics Department and consists of 16 rooms distributed as follows: Women’s Clinic, Family Medicine Clinic, Nurses & Midwives Room, Doctor’s Room, Central Pharmacy, Operating Room, Recovery Room, Correction & Micro Surgery Room, Sterilization Room, Labor and Delivery Room (L&D), administration offices, and 4 inpatient rooms. The second floor consists of an additional 16 rooms distributed as follows: Pediatric clinic, Dental clinic, Accountant office, women’s waiting room, warehouse, nursing office, laboratory and sample collection room, data input room, Pediatrician on-call room, Nursery, reception room, and 2 Pediatric inpatient bedrooms.

In addition to the main medical facility building, a two-story administration and finance/investment building with a basement floor was built adjacent to the hospital. This building contained a coffee shop, a pharmacy, and rental rooms. In addition to the actual construction of the hospital and administrative building, the project also included a plan to procure essential equipment and supplies prior to the hospital’s launch and operation. Basic equipment and supplies included: (a) a water well that was dug at the site to supply the hospital with a sustainable source of water, (b) outpatient department and ER furniture and equipment, (c) initial equipment for the delivery room, (d) an electric generator for both backup and primary power supply, (e) equipment for the operating room(s), (f) central heating system, (g) basic laboratory equipment, and (h) an X-ray machine.

The combination of the main medical facility, administration building, and medical equipment/supplies provided the necessary resources and facilities for essential healthcare services for IDPs including prenatal health services, labor and delivery, postpartum/postnatal care, inpatient and outpatient pediatric and women’s health services, basic dental care, basic pathology/lab services, and general surgery. The hospital was also built in a way that permits the medical staff to deal with additional functions once at full capacity and staffing (see Table 1).

**Table 1:** Brotherhood Medical Center medical services and estimated number of cases per day.

Function	Est. No. of cases
OB/GYN emergency room visits	100/day
Normal deliveries	25/day
Outpatient cases	300/day
Operative cases	10/day
Inpatient cases	25/day

The construction and furnishing took approximately 1 year to finish before launch and operation. All expenses for project construction; equipment, supplies, and furnishings were provided by a group of individual donors which included a group of physicians and some Saudi business men (i.e., no funding from the local government, NGOs, or other organizations was received).

#### Phase II: The Initial Launch and Operational Phase

On September 2014, nearly 1 year after the purchase of land for the project, the BMC opened its doors to patients. The initial launch was limited to providing essential outpatient services, obstetrics and gynecology services, and running its pediatric clinic. The initial staffing of the BMC at launch consisted of three doctors, a nurse, a midwife, an administrative aide, and a housekeeper. The BMC does not have a specific policy regarding patient eligibility, instead accepting all women and children patients who present at its facility, whether seeking urgent or routine care. Staff at the BMC estimate that there were 300 patients per day who visited the facility from the launch date until the commencement of the second operational phase in April 2016 (described below). In addition to accepting patients regardless of condition or status, all services at the BMC were provided free of charge. During this time, donors covered all operational costs and salaries of BMC staff.

#### Phase III: Planned Scale Up During the Second Operational Phase

After the initial launch and operation of the BMC, there was great enthusiasm and strong initial support from donors, with hopes to scale up the project to meet continuing needs of Syrian IDPs. However, since the launch, the BMC has continued to face several financial and operational challenges, including logistical difficulties in transferring and receiving funds/money in Syria (where the clinic is located), lack of qualified medical personnel to run the clinic, and the closing of borders which limited access to medical and humanitarian supplies. Many of these challenges were compounded by the fact that BMC services have simultaneously expanded to meet the increasing needs of families but have also become more difficult to deliver due to the worsening security situation on the ground.

Due to these challenges, the BMC governance committee and leadership group decided to actively seek additional support and cooperation with an international or local sponsor. This search resulted in a strategic partnership with the Syrian Expatriate Medical Association (SEMA), a non-governmental, non-profit organization that operates and manages medical relief projects in Syria, as well as in Lebanon, Jordan, and Turkey (2). Due to this partnership, in April 2016, the hospital was turned over to SEMA for further development and operation. At that time, the OBGYN department began to conduct simple, uncomplicated cases of normal deliveries. SEMA worked to support BMC with required manpower and equipment, resulting in greater access and steady improvement of clinical services on-site.

Currently, the BMC provides a broader array of healthcare services compared with its initial operational phase and has expanded to providing antenatal care, emergency maternal and child care, inpatient and outpatient care for women and children, normal and cesarean section deliveries, and postnatal care for mothers and babies living within a distance of 250 km from the clinic.

The OB/GYN department is now operating at approximately 70% of its capacity after opening access to the clinic’s inpatient and operating room services which started in November 2016 (at the same time cesarean section deliveries started being provided). Illustrating this increase in provisioning of services, from August 2016 to January 2017, the number of vaginal and cesarean deliveries increased by 119% (from 80 in August 2016 to 175 in January 2017) and outpatient visits increased slightly by 9% (from 3,300 in August 2016 to 3,600 in January 2017).

The now operational pediatric department also provides coverage for emergency services, outpatient care, and in February 2017, began to provide inpatient services. Finally, the BMC also operates a dental clinic that provides access for emergency dental needs such as tooth extraction or treatment of acute dental conditions such as carries. In 2017, the estimated number of patients served by the BMC was 4,000 patients per month.

## Discussion

Despite initial success of the BMC in establishing, constructing, launching, and scaling up services through partnership with SEMA, in order for the clinic to reach its full potential in serving critical needs of Syrian IDPs, a number of current and future challenges need to be addressed. First and foremost, the clinic needs a sufficient and consistent supply of medical equipment, supplies and materials to support the vital processes (operating rooms, laboratory, blood bank, intensive care room, pharmacy, and sophisticated equipment such as imaging and sterilization equipment) of the growing array of services and complex health interventions that it delivers. These challenges are commonplace in health conflict situations and represent a formidable constraint to this design of medical humanitarian intervention in comparison to other forms of aid (such as relief services provided by international organizations or international NGOs).

There are also critical infrastructure upgrades that need to be carried out, including securing an ambulance to transport patients to the clinic from the IDP camp and surrounding community, equipping operating rooms to cover all surgical specialties (including Ob/Gyn and Pediatrics), and installing an elevator to allow ease of transport and access to the second floor of the clinic. Arguably equally if not more important to infrastructure is the critical need for appropriate levels of staffing and salary support for BMC healthcare personnel. This includes an immediate need to hire a radiologist on-site and nurses to operate three newborn incubators that are currently not in operation.

As of February 2017, the BMC staffed 8 physicians, 12 nurses, 5 technicians, 9 administrators, and 12 staff related to other duties at a total monthly cost of $22,350. However, to operate at its full capacity, an increase in staff in areas such as midwives, nurses, and technicians, is deemed necessary and would generate an estimated total monthly payroll of $41,000 (see Table 2). Hence, increased funding is needed to ensure adequate access to essential medical supplies, improve facility infrastructure, and ensure salary support for needed scale up of operations. Without additional support for scale up, demand for BMC services will likely continue to outpace its slowly growing capacity, especially as the current Syrian crisis escalates and continues to put significant pressure on existing but devastated Syrian public health systems.

**Table 2:** Workforce and associated monthly costs compared to additional employees needed to operate at full capacity for 2017 (all in US Dollars).

Position title	Number of employees	Total current cost/budget	Additional employees needed to operate at full capacity?
General Manager	1	$1400	–
Deputy Director	1	$1400	–
OBGYN Physicians	3	$1400	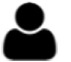
Pediatricians	3	$1200	–
Pediatric Residents	–	–	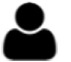 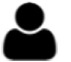 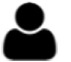
Nurses	8	300	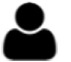 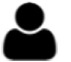
Midwives	4	450	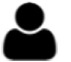 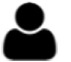
Anesthesia Doctor	1	$1000	–
Surgeon	–	–	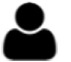
One Anesthetic Technician	1	400	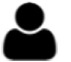 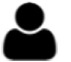
Pharmacist	1	$ 450	–
Pharmacy Technician	–	–	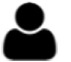 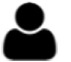 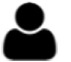
Dentist (dental clinic funded by Idlib Health Directorate)	1	$ 700	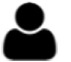
OR Technician	–	–	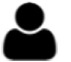 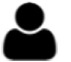 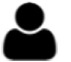
Lab Technician	–	–	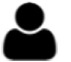 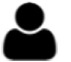 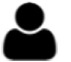
Radiology Technician	–	–	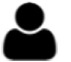 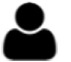
Audiology Technician	–	–	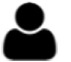
Administrative Supervision	1	$400	–
Administration Staff	6	$300	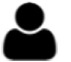 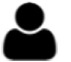 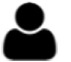 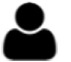
Guard	4	$150	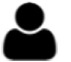 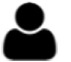
Driver	1	$200	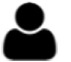 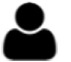
Cook	1	$200	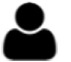
House Keepers	4	$150	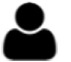 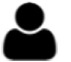 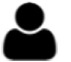 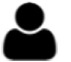 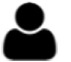 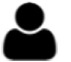
Accountant	1	$ 500	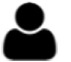
Receptionist	4	$150	–

*Additional Needs: ambulance, elevator, and a radiologist*.

In the long-term, the BMC faces other distinct challenges often faced by medical humanitarian projects that lack direct international support and evolve to continue to serve the needs of a local population that continues to require services as conflicts continue. The first of these challenges is the need to secure sustainable sources of funding beyond initial fund raising and partnerships. Though the BMC has made a first transition to scaling up its services after partnership with SEMA, the resources of Syrian-based organizations like SEMA are under pressure, particularly as the Syrian conflict continues to worsen. Hence, there is a need for the BMC to diversify its funding and partnership networks to other international NGOs and possibly international health and human rights organizations (such as leveraging cash assistance programs operated by UNICEF to subsidize the cost of access to basic health services), particularly as the clinic experiences increases in the number of patients it serves.

Diversification in funding also needs to be accompanied by investment in monitoring and evaluation (M&E) activities to quantify the clinic’s effectiveness and impact and to further enhance its operation and management (including potential use of USAID EONC indicators to establish best practices for high-quality and relevant essential obstetric and newborn care programs) (9). This is particularly important in the context of potential proliferation of NGOs in Syria in a post-conflict phase, which may also lead to an environment that lacks necessary oversight and centralization of humanitarian and health funding. For example, following the 2014 earthquake in Haiti, the country became host to an estimated 10,000 foreign-run NGOs (primarily engaged in projects for reconstruction), yet criticism has been levied over the effectiveness of aid and how NGOs spent these funds. Hence, by developing a robust M&E process, the BMC will be in the position to differentiate itself on the basis of its tangible benefit to its local community now and in the future.

Second, a lack of medical cadre and staffing issues due to the brain drain of skilled workers continues and may not be adequately addressed by securing additional funding. In this case, it may be necessary for the BMC to partner with other medical humanitarian aid groups, such as MSF or Seed Global Health, which can provide temporary staffing and medical professional volunteers. Finally, the factors beyond control of the clinic, including lack of security, poverty, malnutrition, poor sanitation, harsh weather, and lack of proper accommodations for families who continue to reside in temporary shelter, directly impacts the health of BMC’s patient pool, and leads to continued use of services.

Hence, there are important lessons to be learned from the experience of the BMC as a case study in humanitarian medical operations struggling to operate in conflict and war-torn zones. First, despite ongoing challenges, the creation of the BMC shows that the initial actions of individuals can translate into meaningful change and have the potential to be scaled for broader impact. In fact, the BMC intends to carry out a plan for continuation and development, provided external support (either through SEMA or other organizations) can be secured, with the next phase of this project envisioning the construction of a third floor to increase its service capacity. Furthermore, the case study indicates that local and international collaboration can be synergistic, as local groups may be more aware of the acute needs and exact nature of medical humanitarian support required, which can be coordinated with financial and technical support from national and international organizations that are positioned to provide forms of assistance.

## Conclusion

The struggles of women and children enduring the ongoing Syrian health and humanitarian crisis are compounded by severe underfunding of the humanitarian aid response, lack of a sustainable political solution to end the conflict, and the fragmentation of the country into sectarian zones, often out of the reach of humanitarian agencies (10). Although several international NGOs are attempting to meet the needs of IDPs, the acute severity faced by countless Syrian women and children who are the victims of this conflict is impossible to address without targeted interventions specifically designed and deployed where these populations reside. Unfortunately, most Syrian IDPs currently live under harsh, inhumane conditions, a situation worsening with time, especially with continued shrinkage of conflict-free areas. In this context, the BMC represents a unique example of cooperation between local and regional stakeholders with the aim of filling a critical gap in provisioning of medical humanitarian aid. Although the BMC shows promise, the future pathway of this organization requires prioritization and support from the international community recognizing its critical role as a localized medical humanitarian intervention for a population in desperate need of help.

## Ethics Statement

This community case study did not involve the direct participation of human subjects and did not include any personally identifiable health information. Hence, the study did not require ethics approval.

## Author Contributions

We note that with respect to author contributions, all authors jointly collected the data, designed the study, conducted the data analyses, and wrote the manuscript. All authors contributed to the formulation, drafting, completion, and approval of the final manuscript.

## Conflict of Interest Statement

AN and LB were part of the foundation of the BMC clinic and remain active in its operation. The remaining authors declare no conflicts of interest associated with this manuscript.
